# Some Unappreciated Causes of Anemia in Childhood

**Published:** 1903-10-15

**Authors:** W. C. Hollopeter


### SOME UNAPPRECIATED CAUSES OF ANEMIA IN CHILDHOOD.

BY W. C. HOLLOPETER, A.M., M.D.

The time was when the child's physician was called only to the acute troubles of childhood, the chronic sufferer drifted on, handicapped by a host of physical imperfections until an acute disorder would terminate the child's life. In many cases of milder specific diseases the child is left to fight its way back to health, scarcely reaching, unfortunately, its original vigor.

Primary dentition, a physiologic process, is often a time of ill health and suffering to the child, but when this is complicated by the administration of improper food, irregularly given, which sooner or later disturbs the child's balance, we frequently find the little patient markedly anemic at the end of this period.

For the last few years my attention has been constantly drawn to some of the unrecognized, or at least, unappreciated causes of anemia in growing children. We know the anemias of the acute diseases and specific blood changes; of these I do not wish to speak. I have long felt that the gastrointestinal tube was at fault more than the effect of irritating food, and that these conditions might be avoided if the child possessed a clean mouth and if its personal hygiene was all that we could desire.

The great primal cause of anemia in children, and the factor least recognized and yet most prevalent, is dental decay. While the majority of children's physicians are alive to this source of infection, the mass of general practitioners pay but little attention to this important factor, and hence, not until the child becomes markedly anemic and has associated with it, by reason of this lowered resistance many complicating troubles, do they awake to the realization of its importance. Dental decay furnishes more cases of anemia, is more prevalent, is least appreciated of all the disorders of childhood. In a child from six to ten years of age it is a very common thing to find teeth in an advanced state of decay, with a condition of pus formation and general septic involvement of the whole alimentary canal. Too little attention is paid to saving the first teeth. If careful attention is not given to them a general hyperemia of the oral cavity occurs, with numerous alveolar abscesses, which empty their pus contents into the stomach. Bad teeth prevent thorough mastication and in this way contribute materially to a deranged gastrointestinal tract. A general catarrhal inflammation is created that renders the child irritable, provoking a severe trial to his disposition, if not changing his temperament, and thus laying a foundation for anemia which will require years of correct living to outgrow. I have watched a great many children at this age, during the period of second dentition, and have found their mouths, with few exceptions, in a septic condition. This pathologic condition could not exist in any other portion of the body without comment or immediate correction. It goes on, however, in about 90 percent of all children, at this age, uncorrected, unnoticed.

In children with weak digestion and irritable dispositions we are constantly correcting their habits or administering moral suasion instead of removing the pus generating, foul, decaying teeth. The surgeon will wash his hands and sterilize his patient before an operation on the mouth, jaws or stomach, yet at the same time the mouth of this patient will possess numerous pus-producing fountains. An interesting question to be settled by the physician in the future is to what degree the broken health or perverted disposition of children can be traced to decaying teeth with their contributing pus streams to the system.

It has long been a serious question whether gastro-intestinal catarrh could ever be removed. If a septic oral cavity continues for several years we feel quite sure the individual can scarcely regain his usual vigor. A child thus loses the high standard he is entitled to because this essential of his growth is ignored. Nothnagel calls attention to the following trite truth regarding catarrhal inflammation: First a real cure of an acute catarrh can only be brought about through the regenerating processes going on in the organism or the affected tissues. Second, complete recovery is only possible in acute catarrh. The second point is especially emphasized and the assertion is made that a chronic intestinal catarrh is incurable. It is therefore of the utmost importance to prevent the acute catarrh from becoming chronic. This can be accomplished not so much by medication as by careful regulation of diet and regime and careful toilet of the teeth. With carious teeth in early childhood we will have imperfect digestion, and with defective digestion we will soon have gastrointestinal catarrh and its accompanying anemia. Second dentition is fraught with tremendous importance; the rapid development of the whole body, especially the unstable nervous system, makes it a season of great watchfulness and not least among the many clashing elements to engage our attention are the teeth, rapidly undergoing decay and being replaced by new ones. Neglect of a proper toilet of the teeth will bring us stomatitis of every degree of intensity: ulceration, sloughings, gangrene, pyorrhea alveolaris. In the jaws, by extension, we will frequently find periostitis, alveolar abscesses, necrosis; and in the soft parts in the neighborhood of the mouth, by continuity of structure, we find by reason of the septic condition of the mouth, recurrent tonsilitis, pharyngitis, otitis, glandular inflammation, cellulitis, and possibly meningitis by direct extension. The coroner of Philadelphia, a medical man, some years ago informed me in a conversation that fully 50 percent of the cases of death in young children falling under his observation were due to oral sepsis. This contribution to the anemias of childhood by reason of decaying teeth is, in my mind, preventable. Dr. William Hunter, in his admirable essay on oral sepsis, makes this startling yet unappreciated statement, that dental caries is the commonest and most prevalent infection in the body, and that this infection is of a mixed character, including not only the harmless organisms, but also the most active pathogenic blood poisoning organisms, namely, the streptococci and staphylococci.—*Journal of Medico-Chirurgical College.*

